# Delivery of public health interventions by the ambulance sector: a scoping review

**DOI:** 10.1186/s12889-023-16473-2

**Published:** 2023-10-24

**Authors:** Suzanne Ablard, Elisha Miller, Steven Poulton, Anna Cantrell, Andrew Booth, Andrew Lee, Suzanne Mason, Fiona Bell

**Affiliations:** 1 School of Health and Related Research (ScHARR), The University of Sheffield, S1 4DA Sheffield, England; 2 Yorkshire Ambulance Service NHS Trust Headquarters, Springhill 2 Brindley Way, WF2 0XQ Wakefield, England

**Keywords:** Ambulance sector, Public health, Population health

## Abstract

**Background:**

With millions of unscheduled patient contacts every year and increasing call outs clustered around the most deprived communities, it is clear the ambulance sector could have a role to play in improving population health. However, the application and value of a public health approach within the ambulance sector has not been comprehensively explored.

A scoping review was undertaken to explore the role of the ambulance sector in the delivery of public health interventions and what impact this has on population health and ambulance sector outcomes.

**Methods:**

A search strategy was developed on MEDLINE and translated to other major medical and health related bibliographic databases (Embase; CINAHL; HMIC; Science and Social Sciences Citation Index; Cochrane Library) to identify literature published since 2000 in OECD countries. Targeted grey literature, reference list, and citation searching was also carried out.

Search results were downloaded to Microsoft Excel and screened by three reviewers according to pre-determined inclusion / exclusion criteria. Data from included studies, such as the type of activity noted within the paper, the population involved and the public health approach that was utilised, was extracted from within the paper using a data extraction form and narratively synthesised.

**Results:**

Fifty-two references were included in the final review (37 database searching; 9 reference list searching; 6 grey literature).

Included articles were categorised according to the relevant public health domains and subdomains as articulated by the UK Faculty of Public Health:

1. *Health improvement domain:*
  Public health education and advice (Health promotion sub-domain) (*n*=13)
  Emergency Services personnel providing vaccines (Disease prevention sub-domain) (*n*=1)
2. *Health care public health domain*
  Paramedicine (Service delivery sub-domain) (*n*=30)
  Screening tools and referral pathways used by the ambulance sector (Service delivery sub-domain) (*n*=28)
  Health intelligence using ambulance sector data (population health management sub-domain) (*n*=26)
  Of note, some domains (e.g. health protection) returned nil results.

**Discussion:**

The scoping review demonstrates the breadth of public health related activities in which the ambulance sector is involved. However, an overemphasis on demand management outcomes precludes definitive conclusions on the impact of ambulance sector-led public health initiatives on public health outcomes. Future evaluations of public health initiatives should incorporate wider health system perspectives beyond the immediately apparent remit of the ambulance sector.

**Supplementary Information:**

The online version contains supplementary material available at 10.1186/s12889-023-16473-2.

## Background

Historically the primary focus of the ambulance sector has been on the assessment, treatment and/or transportation of acutely unwell patients. With millions of unscheduled patient contacts every year and increasing numbers of call outs often clustered around the most deprived communities [1], it is clear the ambulance sector could have an important role to play in improving population health and wellbeing. However, the public health role and application of a population health approach within the ambulance sector has not been comprehensively explored.

The population health approach aims to improve the health of an entire population, as well as reducing health inequalities [2]. This requires striking a balance between universal service provision and tailored targeted service provision for certain population subgroups. For the ambulance sector this means recognising when a patient presents with a greater level of need, both in terms of their social circumstances and health, and providing an appropriate level of response to support that individual, such as through provision of additional advice or referral to support services [3].

The ambulance sector may also be well situated to address aspects of disease prevention through proactive approaches to prevention, early intervention and reducing crises and subsequent harmful consequences [4]. Opportunities may exist even though the core model for an ambulance service is a reactive response and focused on immediate need, as preventative health advice or support (e.g. smoking cessation, referrals to a falls prevention programme) could be delivered during those patient encounters. These individual patient interactions, when aggregated, also provide a unique perspective on disease burden, and understanding of acuity at a population level.

Population health management approaches are already in use by health service planners. This approach applies data to understand population needs and then informs the design and delivery of care to achieve the best possible health outcomes with the available resources [5, 6]. Ambulance sector data can complement existing systems by providing an additional perspective on disease prevalence, progression and management that may not be captured elsewhere in the system.

As the public health role within the ambulance sector is a relatively new concept, there is limited information about what activities are already taking place, and what the evidence is for the success of such activities. Understanding existing public health activity in this healthcare setting may provide insights into potential future public health approaches. This scoping review of available evidence was undertaken to answer the following research question: *“*(1) *How is the ambulance sector currently involved in the delivery of public health / preventative interventions, and* (2) *what is the impact of this activity on population health and ambulance sector outcomes?”*

## Methods

### Design

A scoping review of published and ‘grey’ literature was undertaken within tight time constraints to answer the research question. Scoping reviews are commonly used to inform decision-making and further research based on the identification and examination of the literature on a given topic or issue [7]. Scoping reviews typically draw on evidence from any research methodology to provide an overview that addresses broader review questions than a focused systematic review. Scoping reviews are not always rapid reviews but often are used to deliver answers within a limited window of opportunity, acknowledged as important for the stakeholders in this project. Although time constraints prohibited conduct of a systematic review, systematic efforts were followed for study identification.

### Search strategy

A sensitive literature search approach was developed to identify published and peer-reviewed literature. The search strategy included thesaurus and free-text terms and relevant synonyms for the population (ambulance services and staff) and intervention (public health / preventative interventions) and used proximity operators where appropriate. The search terms were then combined using Boolean operators.

#### Types of evidence sources

The search strategy was developed on MEDLINE and then translated to the other major medical and health-related bibliographic databases (Embase; CINAHL; HMIC; Science and Social Sciences Citation Index; and the Cochrane Library). The MEDLINE search strategy is provided in the Supplementary material. Outcome terms were not included in the search as this information is not always included in the abstracts meaning that relevant studies might otherwise be missed. The search was limited to research published in English from 2000 to 31st January 2021. An Organization for Economic Cooperation and Development (OECD) filter in development by the UK’s National Institute for Health and Care Excellence (NICE) was used to ensure retrieval of literature from relevant countries [8]. Searches were conducted in February 2021.

Targeted grey literature searches were carried out on websites to identify relevant case studies and reports. A list of the websites searched is provided in the Supplementary material. Reference lists and forward citation searching of included studies were also undertaken to identify additional evidence which may have been missed by topic searches.

### Evidence screening and selection

Eligible studies were selected according to the Population, Concept, Context criteria recommended for a scoping review [7].

#### Population

We included research studies of any study design that reported direct involvement of staff working within the ambulance sector.

#### Concept

We considered involvement in public health as including both interventions and prevention. We referred to published taxonomies of public health practice in order to establish a consistent scope of practice. In cases of uncertainty, brief study details were discussed within the team and, when there was continued ambiguity, a definitive decision was made by a member of the team who is an experienced public health doctor. We excluded studies where staff from the ambulance sector were involved in delivering interventions or prevention within a multi-disciplinary team and the ambulance staff contribution was not distinctly identified. For inclusion, studies had to include outcomes data, whether quantitative or qualitative. Papers that described the intervention without outcomes data were excluded.

#### Context

To be included, a research study had to be conducted within a high-income country where emergency medical systems have similarly developed around an Anglo-American model (using the Organization for Economic Cooperation and Development (OECD) classification) and reported in a publication that was published between 1st January 2000 and 31st January 2021. Studies were limited to those published in the English language. Conceptual papers and studies conducted in low or middle income countries were excluded. Table 1 itemises the inclusion and exclusion criteria.

**Table 1:** Inclusion and exclusion criteria

**Inclusion criteria**	
Date	Evidence published from 1st January 2000–31st January 2021
Setting	Direct involvement by the ambulance service in the intervention
Population	No restrictions on population – included both adult and paediatric studies
Study type	No restrictions on study design but studies must have included outcome data
Model of care	Public health intervention or prevention delivered directly by the ambulance sector
Other	Studies undertaken in high-income countries, published in English language
**Exclusion criteria data were**	
Study type	Papers that describe interventions or services without providing outcome data. Conceptual papers and projected possible future developments
Other	Studies conducted in low or middle income countries

Search results were downloaded to a Microsoft Excel spreadsheet. Three reviewers undertook a pilot study selection exercise which involved a sample of 200 records (100 records each, overlapping each reviewer by 50 records) being independently coded by the individual members of the review team using drop-down menu categories. Discrepancies in the coding were discussed amongst the three reviewers and further questions were raised with the wider project team. The three reviewers then worked independently to code all remaining references retrieved from the database searches. A sample of excluded records for each of the reviewers were screened by a second reviewer to ensure these were not excluded in error. Where a verdict of ‘unsure’ was recorded by one reviewer, these records were passed on to a second reviewer. If no consensus was reached these records were discussed with the wider project team.

Two reviewers undertook data selection of the grey literature. Where a verdict of unsure was reached, these records were passed on to a third reviewer for consensus or were discussed with the wider project team.

**Fig. 1:**
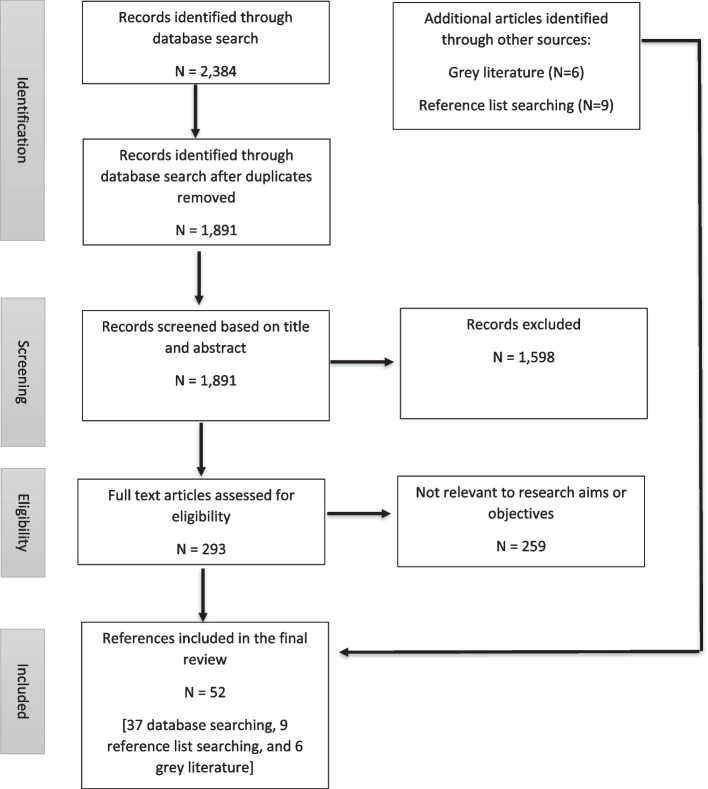
Summary of included and excluded studies

Figure 1 shows the data selection process.

### Data extraction

Data were inputted from included articles into a data extraction form created in Microsoft Excel. Search results were downloaded to Microsoft Excel and screened by three reviewers according to pre-determined inclusion / exclusion criteria. Data from included studies, such as the type of activity carried out, the population involved and the public health approach that was utilised, was extracted from anywhere within the paper using a data extraction form and narratively synthesised. Duplicate data extraction was not possible due to time constraints, but data were iteratively checked and re-checked whilst writing the report. Quality assessment of each study focused on generic limitations of study design only, although specific study design limitations were documented where identified.

### Data analysis

We anticipated that the emerging themes from the review would map to the three recognized domains of public health as articulated by the UK Faculty of Public Health: health protection, health improvement and healthcare public health [8]. The health improvement domain covers both health promotion as well as disease prevention activity. The healthcare public health domain includes aspects of health service delivery and quality such as health data, care pathways and processes. The emergent themes were categorised using this framework. The categorisation of themes was carried out by the lead researcher (SA) and cross-checked by a health subject matter expert (AL) to ensure accurate categorisation.

## Results

### Study characteristics

Thirty-seven studies included in this review were identified through database searching and nine identified through searching the bibliographies of included articles.

Included articles were categorised according to the relevant public health domains and subdomains, as shown in Table 2. Most included studies clustered according to the Healthcare Public Health Domain with some articles also clustering around the Health Improvement Domain, primarily the Health Promotion sub-domain. Of note, some domains (e.g. health protection) had nil returns.

**Table 2:** Categorisation of included papers

Health improvement domain	Number (%)
Health promotion sub-domain: 1. Public health education and advice	6 (13)
Disease prevention sub-domain: 2. Emergency medical services providing vaccines	1 (2)
Healthcare public health domain	
Service delivery sub-domain: 3. Paramedicine 4. Screening tools and referral pathways used by the ambulance sector	14 (30) 13 (28)
Population health management sub-domain: 5. Health intelligence using ambulance sector data	12 (26)

Studies were conducted in 7 countries with 72% undertaken in either the United States of America (USA) or Canada. The target population for most of the interventions evaluated in the studies were adults aged > 18 years.

In all studies evaluating public health education and advice interventions (*n* = 6), the effectiveness of the intervention was assessed using participant self-report measures (e.g., surveys). Four studies used pre-test/post-test measures to assess whether knowledge had increased because of the intervention [9–12]. Two studies collected only post intervention data [13, 14], but one of these studies compared the results to a control group who did not receive the intervention [14].

One study used an observational design to assess the feasibility of emergency medical services providing vaccines [15].

Fourteen studies included in the review described and evaluated paramedicine programmes [16–29]. Seven studies had qualitative designs [17, 24–29], two studies were randomised controlled trials (three papers reported on the same randomised controlled trial) [19–22], one study reported a prospective pre-post intervention [18], and two were relatively small scale (a pilot study and case report) [16, 23].

Thirteen studies described and evaluated screening tools and referral pathways used by the ambulance sector [30–42]. Eleven used observational study designs [30–37, 40–42], and one study (based on two papers) reported a randomised controlled trial [38, 39].

Twelve studies reported on how ambulance sector data can be used for population health monitoring and intervention [43–54]. 2 studies used geocoding methods, 6 studies described data linkage methods, and 2 studies used syndromic surveillance.

Table 3 describes the study characteristics of included studies.

**Table 3:** Study characteristics

Author [Year]	Study design	Country	Population
**Public health education and advice**			
Donohoe et al [2012] [13]	Survey	UK	Members of the public
Hall et al [2002] [9]	Pre-test post-test	USA	Emergency services personnel
Lyngnugaryte-Griksiene et al [2017] [10]	Pre-test post-test	Europe	Emergency services personnel
Meischke et al [2000] [14]	Survey	USA	Adults aged >65 years old
Reeve et al [2008] [11]	Survey	Australia	Paramedics
Tomari et al [2017] [12]	Pre-test post-test	Japan	Children aged 9-10 years and their parents
**Emergency medical services providing vaccines**			
Mosesso et al [2002] [15]	Prospective observational cohort	USA	Adults aged >18 years [eligible for influenza vaccine]
**Paramedicine**			
Agarwal et al [2015] [16]	Pilot study	Canada	Adults aged >65 years
Brydges et al [2016] [17]	Qualitative	Canada	Adults aged >65 years
Agarwal et al [2017] [18]	Prospective pre-post intervention	Canada	Adults aged >65 years
Agarwal et al [2018] [19]	Randomised controlled trial	Canada	Adults aged >55 years
Agarwal et al [2019] [20]	Randomised controlled trial	Canada	Adults aged >55 years
Agarwal et al [2020] [21]	Randomised controlled trial	Canada	Adults aged >55 years
Ashton et al [2017] [22]	Randomised controlled trial	Canada	Adults aged >18 years [and one or more chronic conditions]
Heinelt et al [2014] [23]	Case study	Canada	53-year-old male
Dainty et al [2018] [24]	Qualitative	Canada	Adults aged >18 years and their family
Martin et al [2016] [25]	Observational ethnographic	Canada	Patients and their family / carers
Martin et al [2018] [26]	Observational ethnographic	Canada and USA	Emergency services personnel
Martin-Misener et al [2009] [27]	Qualitative	Canada	Adults aged >18 years
O’Meara et al [2014] [28]	Qualitative	Canada	Patients and health professionals
Patterson et al [2016] [29]	Qualitative and document review	USA	Program leaders
**Screening tools and referral pathways used by the ambulance sector**			
Brice et al [2006] [30]	Retrospective case note review	USA	Adults aged >18 years [new or expectant parents]
Hawkins et al [2007] [31]	Retrospective case note review	USA	Adults aged >18 years [new or expectant parents]
Comans et al [2011] [32]	Observational cross sectional	Australia	Adults aged >65 years [high risk of falls]
Langabeer et al [2020] [33]	Observational	USA	Adults aged >18 years [opioid drug overdose survivors]
Langabeer et al [2020] [34]	Observational	USA	Adults aged >18 years [opioid drug overdose survivors]
Lee et al [2016] [35]	Multi-centre observational	Canada	Adults aged >65 years
Shah et al [2010] [36]	Observational	USA	Adults aged >60 years
Shah et al [2006] [37]	Observational	USA	Adults aged >65 years
Snooks et al [2004] [40]	Observational	UK	Patients meeting criteria to be left at scene by paramedic
Snooks et al [2017] [38]	Randomised controlled trial	UK	Adults aged >65 years [falls related incident]
Snooks et al [2017] [39]	Randomised controlled trial	UK	Adults aged >65 years [falls related incident]
Weiss et al [2003] [41]	Observational	USA	Adults aged >65 years [falls related incident]
Weiss et al [2000] [42]	Observational	USA	Adults aged >18 years [Domestic violence]
**Health intelligence using ambulance sector data**			
Byun et al [2019] [43]	Geocoding methods	USA	Adults aged >65 years [falls related incident]
Coory et al [2009] [44]	Syndromic surveillance	Australia	Routine influenza surveillance in the population
Do et al [2018] [45]	Observational	Canada	Opioid related overdoses
Krafft et al [2003] [46]	Data linkage	Europe	Monitoring emergency medical systems
MacDougall et al [2019] [47]	Data linkage	Canada	Opioid related overdoses
Masho et al [2016] [48]	Observational	USA	Youth violence
McNally et al [2009] [49]	Data linkage	USA	Out of hospital cardiac arrest
McNally et al [2011] [50]	Data linkage	USA	Out of hospital cardiac arrest
Diepen et al [2017] [51]	Data linkage	USA	Out of hospital cardiac arrest
Mears et al [2010] [52]	Data linkage	USA	State-wide emergency medical services data system
Sasaki et al [2010] [53]	Geocoding methods	Japan	Predict future emergency medical service usage
Todkill et al [2017] [54]	Syndromic surveillance	UK	Surveillance of respiratory related illness in the population

Six UK based pilot initiatives were identified through grey literature searching but these had not been rigorously evaluated [55–61]. Table 4 describes the studies identified through grey literature searching.

**Table 4:** Study characteristics – Grey literature

Link	Public health activity	Country	Population
https://aace.org.uk/best-practice/eeast/	Joint initiative with a paramedic and physiotherapist / occupational therapist to provide community health assessments, social services access, equipment provision, and onward referral for health and social support and medication advice.	UK	Older adults.
https://aace.org.uk/best-practice/las/	Paramedic crewed with a mental health nurse, responding to patients requiring a mental health response.	UK	Patients experiencing a mental health crisis.
https://aace.org.uk/initiatives/frailty-response-line-hull-east-riding/	Frailty response line manned by a consultant geriatrician accessible by paramedics and community care staff for frail residents.	UK	Care home patients or patients in their own home with a clinical frailty score of ≥5.
https://aace.org.uk/initiatives/leeds-paramedic-primary-rotation/	Specialist paramedics rotated across 15 GP surgeries conducting home visits. They also rotated through the 999-control centre and provided frontline operational duties to safely manage patients away from the Emergency Department.	UK	Patients registered at 1 of 15 GP surgeries across Leeds or patients across Leeds if visited by a rotational paramedic when on operational duties.
https://aace.org.uk/initiatives/falls-rapid-response-service-frrs/	Paramedic and occupational therapist worked collaboratively to respond to any falls related calls for patients >60 years who had fallen at home.	UK	Adults aged >60 years, living in their own home [care home or sheltered housing included].
https://www.england.nhs.uk/publication/wokingham-paramedic-home-visiting-model/	Integrated paramedic home visiting model with patients being seen at home by a paramedic to reduce GP workload.	UK	All patients registered at Wokingham General Practitioner Alliance PCN.

### Public health education and advice

Two studies from the USA and Japan reported public awareness of preventative health behaviours increased following public health education interventions delivered by emergency medical technicians [12, 14]. However, neither study measured whether participants changed their behaviour in practice nor looked at health care usage post intervention. A UK study [13] followed up their participants to document whether members of the public had acted on advice provided by a paramedic regarding their blood pressure. They found 56% of participants who were advised to contact their GP by the paramedic did so and 42% of participants identified as having high blood pressure by the paramedic reported taking actions to reduce their blood pressure (e.g. by increasing exercise levels). However, the follow-up period was short, making it unclear whether the changes were sustained in the long term and what impact this had on future healthcare usage.

Three studies reported delivering public health training to staff working in the ambulance sector [9–11]. One Australian study [11] reported that after completing the population health component of the Graduate Certificate in Rural and Remote Paramedic Practice, 73% of paramedics said they had changed their practice, 20% had concrete plans to change, and 7% were considering making changes. A European study by Lygnugaryte-Griksiene et al. (2017) [10] delivered suicide-intervention training to emergency medical service providers and found assessment of suicidal risk factors improved six months post training but suicide intervention skills, attitudes towards suicide prevention and strategies for coping with stress remained unchanged. In another study from the USA [9], participants knowledge, attitudes and situational problem solving about domestic violence improved after attending a training session on domestic violence. However, due to a lack of follow-up data in all three studies it is not possible to say whether changes in clinical practice were sustained in the long-term or had a significant impact on the communities in which the paramedics were based.

### Emergency medical services providing vaccines

An observational study, based in the USA, assessed the feasibility of emergency medical service agencies providing influenza vaccines to members of the public [15]. 48% of those who were vaccinated, reported not receiving the influenza vaccine in the previous year, while 34.5% reported that they probably would not have received the vaccine elsewhere if it had not been for the vaccination programme, suggesting emergency medical service agencies were able to reach out to members of the community who may not ordinarily have received the influenza vaccine. However, three-quarters of those vaccinated were 60 years or younger, suggesting further targeting would be needed in the future to reach those most in need of vaccination.

### Paramedicine

Paramedicine programmes aim to increase access to basic primary care and public health services using specially trained emergency medical service providers.

Three studies reported on the CP@clinic [19–21], and three reported on the CHAP-EMS programme [16–18], which were delivered by paramedics to older adults living in low-income housing buildings in Canada. Types of activity included weekly risk assessment, disease prevention, and health promotion sessions. The studies reported emergency service call use was significantly lower in the intervention group and QALY, blood pressure and diabetes risk significantly improved for programme attendees [18–20]. A cost effectiveness analysis of the CP@Clinic found the reduction in emergency service calls as a result of the programme avoided an estimated C$256,583 (~£147.740), which was almost double the cost of implementing the programme in five communities (C$128,462; ~£73,968) [21]. However, the programmes used ‘accommodated’ paramedics who were unable to undertake traditional paramedic duties due to personal limitations (e.g. injury). This approach may not be feasible in other locations.

A randomised controlled trial in Canada was used to evaluate an ‘ageing at home programme’ where paramedics conducted regular home visits and carried out health monitoring of people with chronic conditions who frequently used emergency medical services [22]. Whilst QALY life scores decreased in the intervention and control groups, this effect was lessened in the intervention group. However, a cost-effective analysis based on cost to realise QALY found it was not cost-effective in the long term.

Two North American studies [27, 29], reported positive outcomes related to paramedicine programmes such as reductions in emergency department visits, and hospital admissions and re-admissions but these findings were not based on rigorous evaluation methods.

Key themes from three qualitative studies aiming to understand the experiences of patients and families involved in paramedicine programmes included: [1] Paramedics became a trusted and essential member of the patient’s healthcare team; [2] positive relationships with the paramedics encouraged patients to keep up with positive lifestyle changes and to become more proactive about disease management; [3] paramedics were described as a ‘safety net’, providing a sense of security and support that patients did not have before; [4] long-term improvements in the patient’s health status were reported by paramedics, patients and the patients families [17, 24, 25].

Key themes from two qualitative studies exploring the experiences of paramedics and service managers involved in the delivery of paramedicine programmes included: [1] Community paramedics work in ways that use very different skill sets to the emergency services norm, which can create transitional barriers and role boundary conflicts; [2] Building trust and long term relationships with patients is key to the success of paramedicine programmes; [3] Traditional paramedic training is focused on the emergency medical response, with limited education on health promotion, aged care, and chronic disease management [26, 28].

### Screening tools and referral pathways used by the ambulance sector

Snooks et al. (2017), conducted a cluster randomised controlled trial in the UK evaluating a falls referral pathway [32]. Paramedics in the intervention group only referred 8% of eligible patients but left fewer patients at the scene without any ongoing care compared to paramedics in the control group. Low referral rates were also reported in another study where paramedics only referred 3% of eligible patients to an 8-week fall prevention programme [32]. One reason cited for the low referral rate was that paramedic training is overly focused on acute care and so paramedics may not recognise a non-injurious fall as a clinical incident requiring follow-up care. Another explanation is that patients may be unwilling to be referred because they feel they do not require assistance [36].

Two studies, based in the USA, evaluated an outreach referral service run by paramedics and peer recovery coaches for people who had recently survived an opioid overdose [33, 34]. Of the individuals contacted by the outreach team, 33% engaged in same day treatment and 56% of those were still engaged at 90 days. However, more than half (59%) of potential participants proved unable to be located due to homelessness or living in temporary accommodation.

Two US articles described an intervention where paramedics provided education to new and expectant parents on childhood injury prevention [30, 31]. However, no information was provided about whether the intervention changed behaviour in the long-term or had any impact on subsequent injuries sustained in the home.

Three studies reported on the development and validation of screening tools that were not linked to follow-on services [35, 41, 42]. Whilst the studies reported that screening tools were feasible, the lack of follow-on services means conclusions about whether they impacted on public health or ambulance sector outcomes could not be assessed. Shah et al. (2010), evaluated a screening tool used by paramedics where the results of the screening were sent to the patients GP by the research team [36]. At follow-up the authors reported that infrequent discussions had taken place between the patient and their GP about the risks identified during the screening. However, the 2-week follow-up period may not have been long enough for participants to have received any interventions designed to meet their unmet needs.

A UK based study evaluated the implementation of “treat and refer” protocols which allowed ambulance crews to leave patients at the scene with a referral to community-based services or self-care advice [40]. The median job cycle time was 8 min longer for non-conveyed patients in the intervention group compared to the control group. Whilst the increased time on scene may indicate improved quality of care, this would have a considerable impact on operational performance of an ambulance service.

### Health intelligence using ambulance sector data

Some studies reported using geocoding type methods to identify hotspots within communities where public health interventions may have the greatest impact. For example, Byun et al. (2019) overlaid emergency medical service call data on census tract rates and Google Maps to explore neighbourhoods in Utah (USA) with high fall counts [43]. This information was used by health planners to identify optimal locations for falls prevention programmes in the community. Another USA based data registry reported collecting addresses of cardiac arrest events from 911 call centre data, emergency medical services data and hospital data, enabling the identification of community level disparities related to bystander cardiopulmonary resuscitation and automated external defibrillator use [49–51].

Some studies used data linkage methods to provide a comprehensive picture of population health for use within both regional and country-wide public health initiatives [43, 49, 50, 52]. However, data linkage is highly time consuming as often there is a lack of consistency in reporting methods, and it requires engagement from multiple health agencies [46, 52].

Syndromic surveillance was described in three studies. Syndromic surveillance is the real time collection, analysis, interpretation, and dissemination of health-related data to enable the early identification of the impact of public health threats which requires public health action. Whilst not described as syndromic surveillance, Do et al. (2018), report using ambulance service data to identify opioid-related events in Canada in near real-time [45]. However, one study reported that whilst ambulance sector data had reasonable sensitivity (i.e. it captured most influenza-related events), the data had low specificity (i.e. misidentified some events as influenza-related) [44]. This issue may lead to inaccurate conclusions that could potentially drive strategic decision making.

Data quality and comprehensiveness is frequently cited as a limitation of using ambulance sector data [43–46, 48–52]. For example, ambulance sector data only captures health events where emergency medical services have been notified, resulting in lower rates of detection. Comprehensive data about patient circumstances and system factors is often unavailable. In the future better data linkage between ambulance service data and other data such as primary care may improve the accuracy and quality of data. In turn, this may lead to greater and more effective use of such data for public health.

### Grey literature

Three initiatives involved ambulance sector staff attending the scene of an incident with other health and social care staff such as occupational therapists and physiotherapists, or mental health nurses [55, 56, 59]. All three initiatives reported a reduction in conveyances to the Emergency Department as result of the intervention. Two articles described paramedics who saw patients, identified by their general practitioner (GP) as requiring a home visit, to help reduce GP workload [58, 60]. Reductions were reported in ambulance conveyances, hospital attendances, NHS 111 (urgent) and 999 (emergency) calls, and GP appointments [60]. Furthermore, the proportion of appropriate non-conveyances increased by 35% when paramedics were given the opportunity to work in primary care [58]. Finally, a frailty response line staffed by a consultant geriatrician, accessible to ambulance staff and community care staff to provide care for frail residents reported high proportions of patients being able to remain at home through joint care planning [57]. Whilst the initiatives identified through grey literature searching reported positive outcomes, none of the initiatives had been rigorously evaluated.

## Discussion

The studies included in this review demonstrated the breadth of activities the ambulance sector has been involved in that relate to public health promotion and prevention. However, many interventions focused on demand management (e.g. reduction in ambulance call outs), rather than improvements to population health and wellbeing. As a result, few initiatives reported in the review used rigorous evaluation methods to understand their impact on population health. In addition, longer term public health outcomes require analysis of data across multiple sources which was not reported for studies included in this review. Therefore, it is difficult to draw precise conclusions regarding the broader impacts on population health from included studies. Nevertheless, the review can be used as a starting point for discussions about what role the ambulance sector could have in terms of a public health and prevention agenda in the future.

One area to consider is how the ambulance sector can make every clinical contact count and maximise opportunities to deliver health promotion. This will be contingent on the skills and willingness of the workforce to do so. Paramedics receive initial training in the delivery of acute care services but not public health interventions [32]. It may be that success from public health initiatives has previously been limited by resistance to change [38, 39]. To ensure consistency, the principles of public health and prevention would need to be embedded across the culture of the organisation and would need to include education for all ambulance staff about the determinants of health and wellbeing [11]. However, at present demands for ambulance care mean that expanding the scope of the service may be limited by the need to deliver immediate and emergent care.

Alternatively, a targeted approach may focus on upskilling a selected group of ambulance sector staff with specific remits around public health prevention strategies and who can deliver interventions targeted at specific populations with the greatest need. Community paramedicine is an example of this type of approach in North America [18–29]. Since the paramedics already have an interest in public health, they may be more motivated to build on this in comparison to those with a greater interest in critical care management. However, this approach requires significant commitment by paramedics to undertake the necessary training to expand their scope of practice, and organisational support. In practice, it is likely that a blended approach may be more appropriate with the potential to develop tiered provision of workforce training and education in relation to the public health agenda.

An immediate solution would be to improve data sharing and linkages between ambulance sector data and data collected from other sectors within the health system. The health intelligence garnered would be richer and enable services, policy makers and researchers to identify and target population health needs, or map patient journeys through the entire health system which could help identify where public health initiatives may have the greatest impact.

### Gaps in the evidence base

Perhaps this scoping review is most notable not for what has been uncovered but for its considerable gaps. There is little evidence on interventions specifically focused on disease prevention, risk factor and lifestyle modification, despite the many opportunities ambulance services have to intervene. Little has been published on the potential role of the service tackling wider determinants of health such as poor housing, fuel poverty, air pollution and local employment. Moreover, the current literature does not explore the role and influence of the sector in addressing health inequalities, again despite the fact the ambulance service spans all local populations. It could be that ambulance services are simply not perceived as being the most appropriate for undertaking these roles.

### Limitations

This review was conducted within strict time and resource constraints to meet the needs of the commissioners of this study. Search strategies sought to optimise retrieval of a manageable number of references and therefore other potential sources or studies may not have been identified or retrieved. The review team faced ambiguities with regards to both the conceptualisation of the topic (i.e. what constitutes the scope of public health practice) and its terminology (“emergency services” include but extend beyond ambulance service intervention). However, the team sought to mitigate these challenges through frequent consultation and team meetings and the use of appropriate definitions and conceptual frameworks.

Study selection and data extraction were fast-tracked and typically conducted by one reviewer leading to possible omissions or inconsistencies. However, proportionate quality assurance procedures were followed, and continual checking took place during the write-up phase. Study quality was assessed at study design level and therefore individual studies may have been judged more favourably or less favourably than their generic design might suggest. Initial interpretations were constructed by a single reviewer but taken to the wider team for validation and discussion.

## Conclusion

This scoping review has demonstrated the breadth of public health related activities in which the ambulance sector is involved. However, an overemphasis on demand management outcomes precludes definitive conclusions on the impact of ambulance sector-led public health initiatives on public health outcomes. Future evaluations of public health initiatives should incorporate wider health system perspectives to assess what happens to patients in health sectors beyond the immediately apparent remit of the ambulance sector.

### Supplementary Information

**Additional file 1.**

## Data Availability

The datasets used and/or analysed during the current study are available from the corresponding author on reasonable request.

## Abbreviations

USA: United States of America
GP: General practitioner
