# Supplementary material for: Delivery of public health interventions by the ambulance sector: a scoping review
Source: BMC Public Health. 2023 Oct 24;23:2082. doi: 10.1186/s12889-023-16473-2 (PMC10598948; doi:10.1186/s12889-023-16473-2)
Supplement: Supplementary file 1 — Additional file 1. [file 12889_2023_16473_MOESM1_ESM.docx]

**Supplementary material**

Example search strategy produced in Medline:

Database: Ovid MEDLINE(R) and Epub Ahead of Print, In-Process & Other Non-Indexed Citations and Daily <1946 to February 01, 2021>

Search Strategy:

--------------------------------------------------------------------------------

1 Ambulances/ (6204)

2 ambulance$.ab,kw,ti. (11130)

3 emergency medical technicians/ (5752)

4 emergency medical technician$.ab,kw,ti. (1136)

5 (ems or emt).ab,kw,ti. (37314)

6 paramedic$.ab,kw,ti. (8502)

7 (ambulance adj2 staff).ab,kw,ti. (224)

8 ambulance personnel.ab,kw,ti. (361)

9 Emergency Responders/ (845)

10 first responder$.ab,kw,ti. (2425)

11 or/1-10 (61122)

12 Emergency Medical Services/ (43671)

13 emergency medical service$.ab,kw,ti. (10103)

14 call centers/ or emergency medical dispatch/ or hotlines/ (2972)

15 Air Ambulances/ (2868)

16 telephone support.ab,kw,ti. (724)

17 HEMS.ab,kw,ti. (762)

18 field triage.ab,kw,ti. (274)

19 out-of-hopsital.ab,kw,ti. (1)

20 or/1-10,12-19 (100979)

21 public health/ (83831)

22 public health.ab,kw,ti. (267089)

23 Preventive Medicine/ (11843)

24 (prevent$ adj3 (approach$ or intervention$ or project$ or program$)).ab,kw,ti. (99112)

25 Primary Prevention/ (18908)

26 Secondary Prevention/ (20924)

27 Tertiary Prevention/ (166)

28 Early Medical Intervention/ (3157)

29 ((primary or secondary or tertiary or early) adj3 prevention).ab,kw,ti. (52289)

30 social prescribing.ab,kw,ti. (169)

31 immunization/ or vaccination/ or mass vaccination/ (133375)

32 (vaccin$ or immuni$).ab,kw,ti. (556326)

33 or/21-32 (1033737)

34 Population Health/ (1013)

35 population health.ab,kw,ti. (12867)

36 targeted service provision.ab,kw,ti. (7)

37 (community adj3 defibrillator).ab,kw,ti. (5)

38 or/21-28,34-37 (449525)

39 11 and 33 (2104)

40 20 and 38 (3115)

41 "Organisation for Economic Co-Operation and Development"/ (302)

42 australasia/ or exp australia/ or austria/ or baltic states/ or belgium/ or exp canada/ or chile/ or colombia/ or costa rica/ or czech republic/ or exp denmark/ or estonia/ or europe/ or finland/ or exp france/ or exp germany/ or greece/ or hungary/ or iceland/ or ireland/ or israel/ or exp italy/ or exp japan/ or korea/ or latvia/ or lithuania/ or luxembourg/ or mexico/ or netherlands/ or new zealand/ or north america/ or exp norway/ or poland/ or portugal/ or exp "republic of korea"/ or "scandinavian and nordic countries"/ or slovakia/ or slovenia/ or spain/ or sweden/ or switzerland/ or turkey/ or exp united kingdom/ or exp united states/ (3221766)

43 European Union/ (16427)

44 Developed Countries/ (20680)

45 41 or 42 or 43 or 44 (3236247)

46 39 and 45 (786)

47 limit 46 to english language (706)

48 limit 47 to yr="2000 -Current" (580)

49 40 and 45 (1525)

50 limit 49 to english language (1381)

51 limit 50 to yr="2000 -Current" (1176)

52 51 not 48 (683)

***************************

Search steps 1-10 are thesaurus, free-text and synonyms for the Population facet (ambulance services and staff) these are the terms that are most likely to retrieve relevant literature (most relevant)

Search step 11 combines the Population terms using OR

Search steps 12-19 are broader thesaurus, free-text and synonyms for the Population facet (ambulance services and staff)

Search step 20 combines all the Population terms using OR (most relevant and broader)

Search steps 21-32 are thesaurus, free-text and synonyms for the Intervention facet (public health / preventative interventions) these are the terms that are most likely to retrieve relevant literature (most relevant)

Search step 33 combines the Intervention terms using OR

Search steps 34-37 are broader thesaurus, free-text and synonyms for the Intervention facet (public health / preventative interventions)

Search step 38 combines all the Intervention terms using OR (most relevant and broader)

Search step 39 combines the most relevant Population and Intervention terms together using AND to retrieve literature on how the ambulance sector has been involved in the delivery of public health / preventative interventions

Search step 40 combines all the (most relevant and broader) Population and all the Intervention terms together using AND to retrieve literature on how the ambulance sector has been involved in the delivery of public health / preventative interventions

Search steps 41-45 are OECD countries search filter developed by NICE

Search step 46 combines the most relevant population and intervention with OECD filter

Search step 47 limits the search to literature published in English

Search step 48 limits the search to literature published from 2000-Current

Search step 49 combines all the population and intervention terms with OECD filter

Search step 50 limits the search to literature published in English

Search step 51 limits the search to literature published from 2000-Current

Search step 52 uses NOT this will be for the second stage to remove the literature retrieved in the first stage from the literature retrieved in the second stage and avoid double screening.

**Embase** 1974 to 2021 February 03

| 1. | ambulance/ |
| --- | --- |
| 2. | ambulance$.ab,kw,ti. |
| 3. | rescue personnel/ |
| 4. | emergency medical technician$.ab,kw,ti. |
| 5. | (ems or emt).ab,kw,ti. |
| 6. | paramedical personnel/ |
| 7. | paramedic$.ab,kw,ti. |
| 8. | (ambulance adj2 staff).ab,kw,ti. |
| 9. | ambulance personnel.ab,kw,ti. |
| 10. | emergency responder$.ab,kw,ti. |
| 11. | first responder$.ab,kw,ti. |
| 12. | or/1-11 |
| 13. | emergency health service/ |
| 14. | emergency medical service$.ab,kw,ti. |
| 15. | emergency medical service$.ab,kw,ti. |
| 16. | emergency medical dispatch/ |
| 17. | (hotline$ or call cent$).ab,kw,ti. |
| 18. | air medical transport/ |
| 19. | telephone support.ab,kw,ti. |
| 20. | HEMS.ab,kw,ti. |
| 21. | field triage.ab,kw,ti. |
| 22. | out-of-hospital.ab,kw,ti. |
| 23. | or/1-11,13-22 |
| 24. | public health/ |
| 25. | public health.ab,kw,ti. |
| 26. | preventive medicine/ |
| 27. | (prevent$ adj3 (approach$ or intervention$ or project$ or program$)).ab,kw,ti. |
| 28. | primary prevention/ |
| 29. | secondary prevention/ |
| 30. | prevention/ or tertiary prevention/ |
| 31. | early intervention/ |
| 32. | ((primary or secondary or tertiary or early) adj3 prevention).ab,kw,ti. |
| 33. | social prescribing.ab,kw,ti. |
| 34. | immunization/ or mass immunization/ or vaccination/ |
| 35. | (vaccin$ or immuni$).ab,kw,ti. |
| 36. | or/24-35 |
| 37. | population health/ |
| 38. | population health.ab,kw,ti. |
| 39. | targeted service provision.ab,kw,ti. |
| 40. | (community adj3 defibrillator).ab,kw,ti. |
| 41. | or/24-35,37-40 |
| 42. | 12 and 36 |
| 43. | 23 and 41 |
| 44. | "organisation for economic co-operation and development"/ |
| 45. | australasia/ or exp australia/ or austria/ or baltic states/ or belgium/ or exp canada/ or chile/ or colombia/ or costa rica/ or czech republic/ or exp denmark/ or estonia/ or europe/ or finland/ or exp france/ or exp germany/ or greece/ or hungary/ or iceland/ or ireland/ or israel/ or exp italy/ or exp japan/ or korea/ or latvia/ or lithuania/ or luxembourg/ or mexico/ or netherlands/ or new zealand/ or north america/ or exp norway/ or poland/ or portugal/ or exp "republic of korea"/ or "scandinavian and nordic countries"/ or slovakia/ or slovenia/ or spain/ or sweden/ or switzerland/ or turkey/ or exp united kingdom/ or exp united states/ |
| 46. | European Union/ |
| 47. | developed country/ |
| 48. | or/44-47 |
| 49. | 42 and 48 |
| 50. | limit 49 to english language |
| 51. | limit 50 to yr="2000 -Current" |
| 52. | limit 51 to embase |
| 53. | 43 and 48 |
| 54. | limit 53 to english language |
| 55. | limit 54 to yr="2000 -Current" |
| 56. | limit 55 to embase |
| 57. | 56 not 52 |

[CINAHL via EBSCO](https://www.sheffield.ac.uk/library/cdfiles/cinahlebsco) 1981-

#QueryLimiters/ExpandersLast Run ViaResults

S52 (S41 AND S50)Limiters - Published Date: 20000101-20210231; English Language; Exclude MEDLINE records Expanders - Apply equivalent subjects Search modes - Boolean/Phrase

S51 (S40 AND S50)Limiters - Published Date: 20000101-20210231; English Language; Exclude MEDLINE records Expanders - Apply equivalent subjects Search modes - Boolean/Phrase

S50 S42 OR S43 OR S44 OR S45 OR S46 OR S48 OR S49 Expanders - Apply equivalent subjects Search modes - Boolean/Phrase

S49 (MH "Developed Countries") Expanders - Apply equivalent subjects Search modes - Boolean/Phrase

S48 (MH "European Union") Expanders - Apply equivalent subjects Search modes - Boolean/Phrase

S47 TI ( "scandinavian and nordic countries" ) OR AB ( "scandinavian and nordic countries" ) Expanders - Apply equivalent subjects Search modes - Boolean/Phrase

S46 TI australasia OR AB Australasia Expanders - Apply equivalent subjects Search modes - Boolean/Phrase

S45 (MH "Norway") OR (MH "Poland") OR (MH "Portugal") OR (MH "South Korea") OR (MH "Slovakia") OR (MH "Slovenia") OR (MH "Spain") OR (MH "Sweden") OR (MH "Switzerland") OR (MH "Turkey") OR (MH "United Kingdom+") OR (MH "United States+") Expanders - Apply equivalent subjects Search modes - Boolean/Phrase

S44 (MH "North America") Expanders - Apply equivalent subjects Search modes - Boolean/Phrase

S43 (MH "Australia+") OR (MH "Austria") OR (MH "Baltic States") OR (MH "Belgium") OR (MH "Canada+") OR (MH "Chile") OR (MH "Colombia") OR (MH "Costa Rica") OR (MH "Czech Republic") OR (MH "Denmark") OR (MH "Estonia") OR (MH "Europe") OR (MH "Finland") OR (MH "France") OR (MH "Germany+") OR (MH "Greece") OR (MH "Hungary") OR (MH "Iceland") OR (MH "Ireland") OR (MH "Israel") OR (MH "Italy") OR (MH "Japan") OR (MH "Korea") OR (MH "Latvia") OR (MH "Lithuania") OR (MH "Luxembourg") OR (MH "Mexico") OR (MH "Netherlands") OR (MH "New Zealand") Expanders - Apply equivalent subjects Search modes - Boolean/Phrase

S42 (MH "Organisation for Economic Co-Operation and Development") Expanders - Apply equivalent subjects Search modes - Boolean/Phrase

S41 (S20 AND S39) Expanders - Apply equivalent subjects Search modes - Boolean/Phrase

S40 (S11 AND S34) Expanders - Apply equivalent subjects Search modes - Boolean/Phrase

S39 S21 OR S22 OR S23 OR S26 OR S27 OR S30 OR S31 OR S32 OR S33 OR S35 OR S36 OR S37 OR S38 Expanders - Apply equivalent subjects Search modes - Boolean/Phrase

S38 TI community defibrillator OR AB community defibrillator Expanders - Apply equivalent subjects Search modes - Boolean/Phrase

S37 TI targeted service provision OR AB targeted service provision Expanders - Apply equivalent subjects Search modes - Boolean/Phrase

S36 TI population health OR AB population health Expanders - Apply equivalent subjects Search modes - Boolean/Phrase

S35 (MH "Population Health") Expanders - Apply equivalent subjects Search modes - Boolean/Phrase

S34 (S21 OR S22 OR S23 OR S26 OR S27 OR S30 OR S31 OR S32 OR S33) Expanders - Apply equivalent subjects Search modes - Boolean/Phrase

S33 TI ( vaccin* OR immuni* ) OR AB ( vaccin* OR immuni* ) Expanders - Apply equivalent subjects Search modes - Boolean/Phrase

S32 (MH "Immunization") Expanders - Apply equivalent subjects Search modes - Boolean/Phrase

S31 TI social prescribing OR AB social prescribing Expanders - Apply equivalent subjects Search modes - Boolean/Phrase

S30 (S28 AND S29) Expanders - Apply equivalent subjects Search modes - Boolean/Phrase

S29 TI prevention OR AB prevention Expanders - Apply equivalent subjects Search modes - Boolean/Phrase

S28TI ( primary OR secondary OR tertiary OR early ) OR AB ( primary OR secondary OR tertiary OR early ) Expanders - Apply equivalent subjects Search modes - Boolean/Phrase

S27 (MH "Early Intervention") Expanders - Apply equivalent subjects Search modes - Boolean/Phrase

S26 (S24 AND S25) Expanders - Apply equivalent subjects Search modes - Boolean/Phrase

S25 TI ( approach* OR intervention* OR poject* OR program* ) OR AB ( approach* OR intervention* OR poject* OR program* ) Expanders - Apply equivalent subjects Search modes - Boolean/Phrase

S24 TI prevent* OR AB prevent* Expanders - Apply equivalent subjects Search modes - Boolean/Phrase

S23 (MH "Preventive Health Care") Expanders - Apply equivalent subjects Search modes - Boolean/Phrase

S22 TI public health OR AB public health Expanders - Apply equivalent subjects Search modes - Boolean/Phrase

S21 (MH "Public Health") Expanders - Apply equivalent subjects Search modes - Boolean/Phrase

S20 (S1 OR S2 OR S3 OR S4 OR S5 OR S6 OR S7 OR S8 OR S9 OR S10 OR S12 OR S13 OR S14 OR S15 OR S16 OR S17 OR S18 OR S19) Expanders - Apply equivalent subjects Search modes - Boolean/Phrase

S19 TI out-of-hospital OR AB out-of-hospital Expanders - Apply equivalent subjects Search modes - Boolean/Phrase

S18 TI field triage OR AB field triage Expanders - Apply equivalent subjects Search modes - Boolean/Phrase

S17 TI HEMS OR AB HEMS Expanders - Apply equivalent subjects Search modes - Boolean/Phrase

S16 TI telephone support OR AB telephone support Expanders - Apply equivalent subjects Search modes - Boolean/Phrase

S15 TI ( call center* OR call centre* OR hotline* OR medical dispatch ) OR AB ( call center* OR call centre* OR hotline* OR medical dispatch ) Expanders - Apply equivalent subjects Search modes - Boolean/Phrase

S14 (MH "Emergency Medical Service Communication Systems") Expanders - Apply equivalent subjects Search modes - Boolean/Phrase

S13 TI emergency medical service* OR AB emergency medical service* Expanders - Apply equivalent subjects Search modes - Boolean/Phrase

S12 (MH "Emergency Medical Services") Expanders - Apply equivalent subjects Search modes - Boolean/Phrase

S11 (S1 OR S2 OR S3 OR S4 OR S5 OR S6 OR S7 OR S8 OR S9 OR S10) Expanders - Apply equivalent subjects Search modes - Boolean/Phrase

S10 TI first responder* OR AB first responder* Expanders - Apply equivalent subjects Search modes - Boolean/Phrase

S9 TI emergency responder* OR AB emergency responder* Expanders - Apply equivalent subjects Search modes - Boolean/Phrase

S8 TI ambulance personnel OR AB ambulance personnel Expanders - Apply equivalent subjects Search modes - Boolean/Phrase

S7 TI ambulance staff OR AB ambulance staff Expanders - Apply equivalent subjects Search modes - Boolean/Phrase

S6 TI paramedic* OR AB paramedic* Expanders - Apply equivalent subjects Search modes - Boolean/Phrase

S5 TI ( ems OR emt ) OR AB ( ems OR emt ) Expanders - Apply equivalent subjects Search modes - Boolean/Phrase

S4 (MH "Emergency Medical Technicians") Expanders - Apply equivalent subjects Search modes - Boolean/Phrase

S3 TI ambulance* OR AB ambulance* Expanders - Apply equivalent subjects Search modes - Boolean/Phrase

S2 TI ambulance* OR AB ambulance* Expanders - Apply equivalent subjects Search modes - Boolean/Phrase

S1 (MH "Ambulances") Expanders - Apply equivalent subjects Search modes - Boolean/Phrase

**Cochrane Library**

Search Name: PHE Ambulance review

Last Saved: 05/02/2021 12:23:03

ID Search

#1 MeSH descriptor: [Ambulances] explode all trees

#2 (ambulance*):ti,ab,kw

#3 MeSH descriptor: [] explode all trees

#4 (emergency medical technician*):ti,ab,kw

#5 (ems OR emt):ti,ab,kw

#6 (paramedic*):ti,ab,kw

#7 (ambulance staff):ti,ab,kw

#8 (ambulance personnel):ti,ab,kw

#9 MeSH descriptor: [Emergency Responders] explode all trees

#10 (first responder*):ti,ab,kw

#11 #1 OR #2 OR #3 OR #4 OR #5 OR #6 OR #7 OR #8 OR #9 OR #10

#12 MeSH descriptor: [Emergency Medical Services] explode all trees

#13 (emergency medical service*):ti,ab,kw

#14 MeSH descriptor: [Call Centers] explode all trees

#15 MeSH descriptor: [Emergency Medical Dispatch] explode all trees

#16 MeSH descriptor: [Hotlines] explode all trees

#17 MeSH descriptor: [Air Ambulances] explode all trees

#18 (telephone support):ti,ab,kw

#19 (HEMS):ti,ab,kw

#20 (field triage):ti,ab,kw

#21 (out-of-hospital):ti,ab,kw

#22 #1 OR #2 OR #3 OR #4 OR #5 OR #6 OR #7 OR #8 OR #9 OR #10 OR #12 OR #13 OR #14 OR #15 OR #16 OR #17 OR #18 OR #19 OR #20 OR #21

#23 MeSH descriptor: [Public Health] explode all trees

#24 (public health):ti,ab,kw

#25 MeSH descriptor: [Preventive Medicine] explode all trees

#26 (prevent*):ti,ab,kw

#27 (approach* OR intervention* OR project* OR program*):ti,ab,kw

#28 #26 AND #27

#29 MeSH descriptor: [Primary Prevention] explode all trees

#30 MeSH descriptor: [Tertiary Prevention] explode all trees

#31 MeSH descriptor: [Early Medical Intervention] explode all trees

#32 MeSH descriptor: [Secondary Prevention] explode all trees

#33 (prevention):ti,ab,kw

#34 (primary OR secondary OR tertiary OR early):ti,ab,kw

#35 #33 AND #34

#36 (social prescribing):ti,ab,kw

#37 MeSH descriptor: [Immunization] explode all trees

#38 MeSH descriptor: [Vaccines] explode all trees

#39 MeSH descriptor: [Mass Vaccination] explode all trees

#40 (vaccin* OR immuni*):ti,ab,kw

#41 #23 OR #24 OR #25 OR #28 OR #29 OR #30 OR #31 OR #32 OR #35 OR #36 OR #37 OR #38 OR #39 OR #40

#42 MeSH descriptor: [Population Health] explode all trees

#43 (population health):ti,ab,kw

#44 (targeted service provision):ti,ab,kw

#45 (community defibrillator):ti,ab,kw

#46 #23 OR #24 OR #25 OR #28 OR #29 OR #30 OR #31 OR #32 OR #35 OR #36 OR #37 OR #38 OR #39 OR #40 OR #42 OR #43 OR #44 OR #45

#47 #11 AND #41

#48 #22 AND #46

#49 MeSH descriptor: [Organisation for Economic Co-Operation and Development] explode all trees

#50 MeSH descriptor: [Australasia] explode all trees

#51 MeSH descriptor: [Australia] explode all trees

#52 MeSH descriptor: [Austria] explode all trees

#53 MeSH descriptor: [Baltic States] explode all trees

#54 MeSH descriptor: [] explode all trees

#55 MeSH descriptor: [Canada] explode all trees

#56 MeSH descriptor: [Chile] explode all trees

#57 MeSH descriptor: [Colombia] explode all trees

#58 MeSH descriptor: [Costa Rica] explode all trees

#59 MeSH descriptor: [Czech Republic] explode all trees

#60 MeSH descriptor: [Denmark] explode all trees

#61 MeSH descriptor: [Estonia] explode all trees

#62 MeSH descriptor: [Europe] explode all trees

#63 MeSH descriptor: [Finland] explode all trees

#64 MeSH descriptor: [France] explode all trees

#65 MeSH descriptor: [Germany] explode all trees

#66 MeSH descriptor: [Greece] explode all trees

#67 MeSH descriptor: [Hungary] explode all trees

#68 MeSH descriptor: [Iceland] explode all trees

#69 MeSH descriptor: [Ireland] explode all trees

#70 MeSH descriptor: [Israel] explode all trees

#71 MeSH descriptor: [Italy] explode all trees

#72 MeSH descriptor: [Japan] explode all trees

#73 MeSH descriptor: [Korea] explode all trees

#74 MeSH descriptor: [Latvia] explode all trees

#75 MeSH descriptor: [Lithuania] explode all trees

#76 MeSH descriptor: [Luxembourg] explode all trees

#77 MeSH descriptor: [Mexico] explode all trees

#78 MeSH descriptor: [Netherlands] explode all trees

#79 MeSH descriptor: [New Zealand] explode all trees

#80 MeSH descriptor: [North America] explode all trees

#81 MeSH descriptor: [Norway] explode all trees

#82 MeSH descriptor: [Poland] explode all trees

#83 MeSH descriptor: [Portugal] explode all trees

#84 MeSH descriptor: [Republic of Korea] explode all trees

#85 MeSH descriptor: [Scandinavian and Nordic Countries] explode all trees

#86 MeSH descriptor: [Slovakia] explode all trees

#87 MeSH descriptor: [Slovenia] explode all trees

#88 MeSH descriptor: [Spain] explode all trees

#89 MeSH descriptor: [Sweden] explode all trees

#90 MeSH descriptor: [Switzerland] explode all trees

#91 MeSH descriptor: [Turkey] explode all trees

#92 MeSH descriptor: [United Kingdom] explode all trees

#93 MeSH descriptor: [United States] explode all trees

#94 (belgium):ti,ab,kw

#95 MeSH descriptor: [European Union] explode all trees

#96 MeSH descriptor: [Developed Countries] explode all trees

#97 #49 OR #50 OR #51 OR #52 OR #53 OR #55 OR #56 OR #57 OR #58 OR #59 OR #60 OR #61 OR #62 OR #63 OR #64 OR #65 OR #66 OR #67 OR #68 OT #69 OR #70 OR #71 OR #72 OR #73 OR #74 OR #75 OR #76 OR #77 OR #78 OR #79 OR #80 OR #81 OR #82 OR #83 OR #84 OR #85 OR #86 OR #87 OR #88 OR #89 OR #90 OR #91 OR #92 OR #93 OR #94 OR #95 OR 96

#98 #47 AND #97

Science Citation Index and Social Sciences Citation Index Web of Science Core Collection, 1900-present

**#1 TS=(ambulance* OR "emergency medical technician*" OR ems OR emt OR paramedic* OR "ambulance staff" OR "ambulance personnel" OR "emergency responder*" OR "first responder*")**

**#2 TS=("emergency medical service*" OR "call center*" OR "call centre*" OR "emergency medical dispatch" OR hotline* OR "telephone support" OR HEMS OR "field triage" OR "out-of-hospital")**

**#3 #2 OR #1**

**#4 TS=("public health" OR "preventive medicine" OR "prevent* approach*" OR "prevent* intervention*" OR "prevent* project*" OR "prevent* program*" OR "primary prevention" OR "secondary prevention" OR "tertiary prevention" OR "early prevention" OR "social prescribing" OR vaccin* or immuni*)**

**#5 TS=("population health" OR "targeted service provision" OR "community defibrillator")**

**#6 #5 OR #4**

**#7 #4 AND #1**

**#8 #6 AND #3**

**#9 TS=("Organisation for Economic Co-Operation and Development" OR australasia or australia or austria or baltic states or belgium or canada or chile or colombia or costa rica or czech republic or denmark or estonia or europe or finland or france or germany or greece or hungary or iceland or ireland or israel or italy or japan or korea or latvia or lithuania or luxembourg or mexico or netherlands or new zealand or north america or norway or poland or portugal or "republic of korea" or "scandinavian and nordic countries" or slovakia or slovenia or spain or sweden or switzerland or turkey or united kingdom or united states or European Union or Developed Countries)**

**#10 #9 AND #7**

**#11 #9 AND #8**

**#12 #9 AND #7 LANGUAGES: (ENGLISH)**

**#13 #9 AND #7 LANGUAGES: (ENGLISH) AND PUBLICATION YEARS: (2021 OR 2013 OR 2005 OR 2020 OR 2012 OR 2004 OR 2019 OR 2011 OR 2003 OR 2018 OR 2010 OR 2002 OR 2017 OR 2009 OR 2001 OR 2016 OR 2008 OR 2000 OR 2015 OR 2007 OR 2014 OR 2006)**

**#14 #9 AND #8 LANGUAGES: (ENGLISH)**

**#15 #9 AND #8 LANGUAGES: (ENGLISH) AND PUBLICATION YEARS: (2021 OR 2013 OR 2005 OR 2020 OR 2012 OR 2004 OR 2019 OR 2011 OR 2003 OR 2018 OR 2010 OR 2002 OR 2017 OR 2009 OR 2001 OR 2016 OR 2008 OR 2000 OR 2015 OR 2007 OR 2014 OR 2006)**

Targeted grey literature searches were carried out to identify reports / case studies in the following websites:

- The Nuffield Trust <https://www.nuffieldtrust.org.uk/>
- The Health Foundation <https://www.health.org.uk/>
- Department of Health and Social Care <https://www.gov.uk/government/organisations/department-of-health-and-social-care>
- NIHR Journals Library <https://www.journalslibrary.nihr.ac.uk/#/>
- NHS England <https://www.england.nhs.uk/>
- Ambulance Service Trusts in the UK individual websites from list at: <https://aace.org.uk/uk-ambulance-service/>
- Association of Ambulance Chief Executives <https://aace.org.uk/>
- Joint Royal Colleges Ambulance Liaison Committee (JRCALC) <https://www.jrcalc.org.uk/>
- College of Paramedics <https://www.collegeofparamedics.co.uk/>
- Royal College of Emergency Medicine <https://www.rcem.ac.uk/>
- Primary Care Networks – websites of individual networks located through internet search on https://www.google.com/
- A&E Delivery boards - websites of individual boards located through internet search on <https://www.google.com/>

Studies identified through database searching and bibliographies of included studies:

| **Author [Year]** | **Description of public health activity** | **Summary of findings [Limitations]** |
| --- | --- | --- |
| **Public health education and advice** | | |
| Donohoe et al [2012] | “Know your blood pressure campaign”: Members of the public offered free blood pressure testing by paramedic, demonstration of ‘face, arm, speech (FAST) used to identify stroke, and given leaflet about risks associated with high blood pressure and benefits of a healthy lifestyle. Members of the public with high blood pressure readings were referred for further medical assessment. | 2274 members of the public had their blood pressure measured. 522 (23%) were referred for further medical assessment. Of those referred, 68% were previously unaware of any blood pressure problems, 56% contacted their GP as advised, and 42% said they had taken actions to reduce their blood pressure. 73% of those surveyed said their awareness of the risks of high blood pressure had increased because of the campaign. [No long-term follow-up so unclear whether behavioural changes were sustained] |
| Hall et al [2002] | Emergency medical service personnel undertook domestic violence training. | 33 emergency medical services personnel undertook the training. Of the 12 items testing participants knowledge, attitudes, and situational problem solving, 11 showed improvement after the training. Participants reported concerns about their preparedness for handling domestic violence calls and supported further training on scene safety, legal considerations, documentation, and community referral. [No follow-up to assess whether the training impacted clinical practice] |
| Lyngnugaryte-Griksiene et al [2017] | Emergency medical service personnel undertook suicide prevention-intervention training. | 268 completed the pre-test survey and 226 completed the post-test survey. Six months after completing the training assessment of suicidal risk factors had improved but suicide intervention skills, attitudes towards suicide prevention, and strategies of coping with stress were unchanged. [No follow-up to assess whether the training impacted clinical practice] |
| Meischke et al [2000] | A “heart attack survival kit” aiming to educate older adults about how to respond to a heart attack was delivered face to face by an emergency medical technician or via the post. | 705 completed the survey – 268 from the emergency medical technician (EMT) delivery group, 214 from the post group, and 223 from a control group who did not receive the heart attack survival kit. Respondents in the intervention group (EMT delivery and post group combined) reported a greater frequency of the recommended coping response to acute myocardial infarction (AMI) (39%) than respondents in the control group (10%) (P<0.000). Within intervention groups, 47% in EMT group and 30% in the post group (P<0.000) reported intentions to take the appropriate response to AMI. [Did not obtain outcome data (e.g. actual 911 calls or aspirin use) therefore relied on self-reported intentions as a proxy for actual behaviour during a AMI]. |
| Reeve et al [2008] | Paramedics undertook the population health component of the Graduate Certificate in Rural and Remote Paramedic Practice which focused on health promotion and chronic disease management. | 20 students completed the pre-course survey and 16 complete the post-course survey. As a result of undertaking the course, 73% of students have already changed their practice, 20% have concrete plans to change and 7% were considering making changes. Changes included increased health promotion and education, improved partnerships with other healthcare providers in the community, and greater engagement in service provision and patient care. [Reliant on self-report intentions with no follow-up data to assess the impact of training on clinical practice and patients within the community]. |
| Tomari et al [2017] | Emergency Medical Technicians delivered a lesson to elementary school children which aimed to increase awareness of stroke. Children were provided with educational materials to share with their parents. | 763 children and 489 parents were included in the analysis. Awareness of stroke symptoms and risk factors immediately after the lesson and 3 months later were significantly higher than those before the lesson both in children and the parents (p<0.01). [Did not obtain outcome data (e.g. number of patients transferred to stroke unit) therefore relied on self-report measures as a proxy for actual behaviour during a stroke]. |
| **Emergency medical services providing vaccines** | | |
| Mosesso et al [2002] | “MEDICVAXX project” – Emergency medical services administered influenza vaccines to members of the public. | 48% of people vaccinated by a paramedic reported not receiving an influenza vaccine in the previous year and 34.5% said they probably wouldn’t have received the vaccine elsewhere without the MEDICVAXX project. Service managers reported the vaccine programme did not impact normal emergency services operations. [The authors noted the most vulnerable adults (over 65 years) constituted a small proportion of the overall number of people vaccinated by a paramedic. Therefore, a more targeted approach in the future would be needed to reach the most vulnerable]. |
| **Paramedicine programmes** | | |
| Agarwal et al [2015]; Brydges et al [2016]; Agarwal et al [2017] | “Community Health Assessment Program through Emergency Medical Services (CHAP-EMS)”: Weekly cardiovascular and diabetes health prevention programme delivered by paramedics. | Agarwal (2015): During the 1-year pilot phase 79 out of 234 eligible participants engaged in the programme with 48 (25.2%) having 2 or more visits to the program.  Brydges (2016): 15 semi-structured interviews and 10 CHAP-EMS sessions observed. Three themes emerged – (1) Trust care and respect; (2) Paramedics as health advocates; (3) Added value of emergency medical service skills.  Agarwal et al (2017): After one year of implementation CHAP-EMS was associated with a reduction in emergency calls, a reduction in participant blood pressure and a tendency towards lowered diabetes risk.  [Paramedics running the programme were ‘accommodated’ paramedics who were unable to undertake traditional paramedic duties due to pregnancy or injuries. This led to variability in the availability of staff to run the CHAP-EMS programme]. |
| Agarwal et al [2018]; Agarwal et al [2019]; Agarwal et al [2020] | “CP@Clinic”: Paramedics led weekly risk assessment, disease prevention, and health promotion sessions. | Agarwal (2018): 4 intervention and 4 control buildings. Average participation rate for CP@clinic in intervention buildings was 37.6% (n=171), ranging from 14% to 52% across buildings. Agarwal et al (2019): 15 intervention and 15 control buildings. Building participation rates ranged from 10% to 82%. During the intervention period 794 residents attended the CP@clinic, 715 attended at least twice and 644 at least three times. In both studies emergency service call use was significantly lower in the intervention compared to control group. QALY, blood pressure and diabetes risk also significantly improved among CP@clinic attendees.  Agarwal et al (2020) conducted a cost effectiveness analysis and found the reduction in emergency service calls avoided an estimated $C256,583.  [Consistency provided by having the same staff member was beneficial, but this may be difficult to achieve outside the context of this study]. |
| Ashton et al [2017] | “Aging at home programme”: Paramedics worked with local health and care agencies to conduct regular home visits and monitoring of patient’s chronic health conditions. | 200 patients were recruited (100 in the intervention and 100 in the control group). Quality of life scores decreased for both the intervention and control groups, but the reduction was lessened for the intervention group. [The intervention was focused on a specific and complex patient population which had unique challenges, so the findings may not be comparable in other contexts]. |
| Heinelt et al [2014];  Dainty et al [2018] | “Expanding paramedicine in the community (EPIC)” is a regional community-based chronic disease management programme run by paramedics. | Heinelt (2014) – Frequent contact with the patient by the paramedic led to the identification of heart abnormalities which if left untreated could have resulted in myocardial infarction.  Dainty (2018) – Observed and interviewed 30 patients and 10 family members. Four themes were identified: (1) patients reported positive health gains; (2) relationship with paramedic encouraged patient to keep up with positive lifestyle and behaviour changes and to be proactive about disease management; (3) Community paramedics were described as a ‘safety net’; (4) Paramedics became trusted, essential members of the patients healthcare team. [The amount of time needed to train paramedics was noted as a limitation of the programme. There is potential for resistance to change from health care providers, paramedics or patients with regards to paramedics utilising non-traditional roles within primary care]. |
| Martin et al [2016] | Community paramedicine program providing prevention and health maintenance services to assist people to remain living independently at home with appropriate health and social service supports. | 14 interviews. Three themes were identified: (1) Improved health monitoring and primary health care access close to home (2) improved sense of security and support for vulnerable residents in the community (3) improved education and empowerment for better health management [The authors noted acceptance of the community paramedicine programme may have been facilitated by residents previously having limited choice or access to primary health monitoring] |
| Martin et al [2018] | Two rural community paramedicine programs designed to provide health monitoring, health education, and early intervention / prevention | 37 stakeholders participated in the focus groups. Paramedics were attracted to the innovative nature of the role, providing preventative care options for patients. Transitional challenges included navigating untraditional roles and role boundary tensions between disciplines. |
| Martin-Misener et al [2009] | A rural primary healthcare model where adults received primary health care and emergency services from an on-site nurse practitioner and paramedic, and an off-site physician. | 86 participants year one, 85 at year two, and 50 at year three. Over the duration of the study emergency department visits decreased. Health promotion, early detection, screening and disease management services increased [Not clear how involved the paramedics were in the delivery of the service (e.g. number of visits to patients)] |
| O’Meara et al [2014] | “Renfrew community paramedic programme” which consists of four elements: (1) Ageing at home program; (2) Paramedic wellness clinics; (3) Ad hoc home visiting programme; (4) Paramedic response unit program. | 3 focus groups and 11 interviews. Key themes: (1) Community paramedics identified they were working in ways that used very different skill sets from the emergency medical services norm; (2) Building trust and long term relationships were seen as enabling factors underpinning the emerging role of community paramedicine; (3) Traditional paramedicine education programs are narrowly focused on emergency response, with limited education in health promotion, aged care and chronic disease management. |
| Patterson et al [2016] | 32 rural serving community paramedicine programmes across the USA. | Outcome data was provided for 13 out of 32 programmes included in the study. Significant reductions were reported in hospital admissions or readmissions (8 programs), health care costs (8 programs), emergency medical services use (6 programs) and emergency department visits (5 programs). [Only a few of the programs which included outcome data had used rigorous evaluation methods]. |
| **Screening tools and referral pathways used by the ambulance sector** | | |
| Brice et al [2006]; Hawkins et al [2007] | “Infant safe homes injury prevention programme” – Paramedic visited the homes of new and expectant parents, conducted a home safety checklist to identify specific hazards, and provided education about childhood injury prevention. | Brice [2006] – 110 home visits were evaluated and only 24 of these were deemed ‘infant safe’.  Hawkins [2007] – 262 home visits were evaluated. High rates of fire and burn hazards and unsafe storage practices.  [No follow-up data documenting changes in home safety practices or incidence of injuries in children in participating households]. |
| Comans et al [2011] | Paramedic referral to an 8 week falls prevention programme led by a community rehabilitation service. | Within 2 years only 17 referrals had been made to the referral service which was equivalent to 3% of the population who could have been referred. Of those who were referred, many were inappropriate for the service. [The authors noted one explanation for the low referral rates is that paramedic training is overly focused on acute care meaning paramedics may not recognise a non-injurious fall as a clinical incident requiring follow-up care]. |
| Langabeer et al [2020];  Langabeer et al [2020] | “Houston Emergency Opioid Engagement System [HEROES]” – Home-based community outreach by paramedics and peer recovery coaches aiming to engage people who have recently experienced and opiod overdose into treatment. | 103 individuals were contacted and screened. 34 (33%) chose to enter the program. Retention in the treatment program was 88% after 30 days and 56% after 90 days. [The outreach team were unable to locate 59% of those screened as being eligible for outreach. Many individuals who overdose are homeless or living in temporary accommodation. Therefore, whilst first responder data captured the location of the overdose, this may be different to their home address. No follow-up data on subsequent healthcare use following engagement with the HEROES program]. |
| Lee et al [2016] | “Paramedics assessing elders at risk for independence loss (PERIL)” – Clinical prediction tool designed to assess whether an adult is at high risk for repeated ED visits, hospitalisation, or death in the month following an emergency services encounter. | Paramedics screened 1,418 older adults. The predictive performance of the PERIL tool was moderate, but the authors noted that it was designed to maximise specificity over sensitivity to avoid patients being falsely identified as being at high risk. Paramedics were more likely to complete observation-based items as opposed to items that require them to ask patients additional questions outside of their usual workflow (e.g. previous 911 use). |
| Shah et al [2010] | Paramedics screened participants across three domains: falls, depression, and medication management strategies. Results were then reviewed by 2 case managers (nurse and social worker) who offered a home visit to those identified as having additional needs. | Paramedics completed at least part of the screening instrument on 85% of visits. Depression was screened successfully on 59% of visits; falls in 66% of visits; and medication management strategies in 77% of visits. Many participants refused the in-home assessment by the case mangers because they did not feel they needed assistance or because they could not be contacted for the assessment. [Improvement on health outcomes for those accessing the program was not measured]. |
| Shah et al [2006] | Emergency medical technicians screened adults aged 65 years and older to evaluate risk of falls, need for pneumococcal vaccine, and need for influenza vaccine. Screening information was sent to study staff who notified the GP of at-risk patients. | 80% of intervention group participants were successfully screened. Participation in the screening led to statistically higher pneumococcal vaccination rates compared to participants in the control group who had not been screened but no differences were reported in influenza vaccine rates or changes to prevent falls. Of those who had been screened there was poor recollection of receiving educational materials and infrequent discussions with GPs regarding their risks. [The follow-up period was 2 weeks which may not have been long enough for patients to have received any interventions designed to meet their unmet needs]. |
| Snooks et al [2004] | Development and testing of treat and refer protocols for paramedics which allowed them to leave patients at the scene with referral to community-based services or self-care advice. | 251 patients in the intervention group and 537 in the control group. Treat and refer protocols did not increase the number of patients left at the scene. Median job cycle time was 8 minutes longer for non-conveyed patients in the intervention group than in the control group. [Increased time spent on scene would have a considerable impact on operational performance of an ambulance service]. |
| Snooks et al [2017];  Snooks et al [2017] | Support and assessment for fall emergency referrals (SAFER) 2 trial – Pathway enabling paramedics to refer older adults to a community-based falls service following a fall rather than transporting them to ED. | 105 paramedics based at 14 intervention stations attended 3,073 eligible patients. Intervention paramedics referred only 8% of eligible patients to falls services but left fewer patients at the scene without ongoing care. |
| Weiss et al [2003] | Screening tool to assess adults aged >65 years who had fallen. | 70 prospective elderly fall cases were evaluated over a 4 month period and 74 retrospective charts were reviewed over a 2-month period. Data collected prospectively was more accurate compared to data collected retrospectively. [There were no follow-on community services to address unmet needs identified through the screening] |
| Weiss et al [2000] | Domestic violence scene assessment screen (DVSAS) – A screening tool to assess whether a domestic environment had a high risk of domestic violence. | 43 patient transfers. Emergency medical technicians were able to complete the DVSAS at the end of the patient journey with good agreement with the independent observer who completed the DVSAS at the scene. [Following screening, patients were not transferred to any services which could reduce their risk of domestic violence related incidents] |
| **Health intelligence using ambulance sector data** | | |
| Byun et al [2019] | Extracted emergency medical service call data of all fall-related incidents from the Utah prehospital reporting system. Fall-injury points were overlaid on census tract rates and Google Maps to explore neighbourhoods with high fall counts. | The emergency medical service call data was used to identify the optimum locations for falls prevention programmes. This data can help planners decide where best to place public health interventions to have the greatest impact. [There was incomplete and missing data in the Utah prehospital reporting system]. |
| Coory et al [2009] | Assessed whether ambulance dispatch data could be used as an influenza and influenza-like illness syndromic surveillance system. | Routine surveillance for influenza and influenza like illness can provide estimates of the relative severity of influenza seasons. Ambulance dispatch data displayed seasonal trends like those observed in existing influenza-like surveillance systems. Ambulance dispatch data has a wide reach within the community and so has the potential to identify cases not identified in other surveillance systems. [Ambulance dispatch data had a high baseline rate in months when there is usually almost no influenza activity in the community. This suggests the data has many false positives (i.e. misidentified some events as influenza related) but few false negatives (i.e. captured most influenza related events). Therefore it is difficult to know using this data whether early signals were false positives or an early warning of an epidemic year.] |
| Do et al [2018] | Determine whether Ottawa Paramedic Service Data can be routinely used to monitor opioid related overdoses in the community. | The data is available within 24 hours of an emergency event. This means the data can be used for near real time monitoring of opioid related events within a community, prompting public health action when necessary. The data was also geocoded to enable hotspots to be identified. [A lack of consistency, accuracy, and precision in data quality between different jurisdictions made national comparisons difficult; Not all opioid related overdoses are captured by paramedic data; There is a potential for opioid related events to be misclassified.] |
| Krafft et al [2003] | European Emergency Data project designed to create a common framework for monitoring and assessing emergency medical service systems throughout the European Union. | Once established the data will be used to monitor and analyse emergency demand or health care utilisation between countries and within populations to identify hot spots where public health interventions may have the greatest impact. [Design and method of access of emergency medical services differs across Europe creating obstacles when trying to link the different health systems into a cohesive dataset.] |
| MacDougall et al [2019] | Development of the Provincial Overdose Cohort which linked 9 public health surveillance and administrative healthcare data sources [including ambulance service data] for individuals who have experienced opioid-related overdoses. | The cohort can identify risk factors, inform public health interventions, and evaluate response actions. Includes geocoded data to enable hotspots to be identified. The inclusion of multiple data sources provides a more complete understanding of the extent opioid related incidents than any single dataset alone. [Integrating data sets is time consuming and complex, meaning the data cannot be used in real time for public health monitoring.] |
| Masho et al [2016] | Ambulance sector data was used to assess the impact of interventions [e.g. restricted alcoholic beverage licenses] on ambulance call out rates in areas where violence rates are high. | Use of ambulance service data provides the Youth Violence Prevention Centres with data that enables them to assess the trends of more serious violent crime in the community. [Ambulance data only represents injuries that require immediate medical attention; Data collected by multiple ambulance providers often lacks uniformity.] |
| McNally et al [2009]; McNally et al [2011]; Diepen et al [2017] | Cardiac arrest registry to enhance survival (CARES) which collects data to improve the quality of care and outcomes of people who experience an out of hospital cardiac arrest. | Medical directors and public health professionals use CARES data to measure and improve the quality of prehospital care for persons experiencing out of hospital cardiac arrest. Ability to compare performance at the local, regional, and national level. CARES records the address of a cardiac arrest event enabling the identification of community level disparities related to bystander CPR and AED use. [CARES cannot capture out of hospital cardiac arrest events when 911 or emergency medical services are not notified resulting in a lack of detection of some hospital cardiac arrest events. Comprehensive data about patient circumstances and system factors is not available (e.g. quality of CPR)] |
| Mears et al [2010] | The North Carolina Emergency Medical Services data system – Development of a fully integrated state-wide emergency medical services data system to identify, evaluate, target, and improve emergency medical service delivery and patient care. | Linkage of emergency medical services across the whole of North Carolina giving a more comprehensive picture which can be used for both regional and state-wide public health initiatives. [It took the project team 3 years to get full engagement from all emergency medical services agencies. Local emergency medical services needed to move to electronic patient care reporting systems before data could be included in the repository. Significant difference in the data quality and completeness from one emergency medical service agency to another.] |
| Sasaki et al [2010] | Used genetic algorithms to identify optimal ambulance locations based on predicted future emergency medical service cases and to develop future emergency medical service management strategies based on predicted demands. | The data management approach described in this study can be used to support planning decisions about the reallocation of ambulances to optimal locations therefore having the potential to improve service delivery and health outcomes. [The study used census data in conjunction with emergency services data. The authors noted the use of census data may result in the overestimation of emergency medical services future demands since overall population was estimated to decrease. Re-allocation of services was based on road network distances, but this does not account for traffic density patterns in time and space.] |
| Todkill et al [2017] | Determine the feasibility of using ambulance data as part of routine syndromic surveillance activities of respiratory related illness. | A daily automated feed of ambulance data from one large ambulance trust was established. This data was used for syndromic surveillance which can provide early warning of increasing disease activity which may require public health intervention. [Ensuring effective collaboration from service providers was critical with regards to the development and operation of the syndromic surveillance system.] |

Grey literature:

| **Link** | **Description of public health activity** | **Outcomes** |
| --- | --- | --- |
| <https://aace.org.uk/best-practice/eeast/> | Joint initiative with a paramedic and physiotherapist / occupational therapist to provide community health assessments, social services access, equipment provision, and onward referral for health and social support and medication advice. | The car attended 256 people who had suffered falls and gave advice and guidance to other crews on 158 other occasions. The car saved 370 ambulance attendances, 285 conveyances to hospital, 255 ED attendances, 78 unplanned admissions to an acute hospital and 708 bed days. |
| <https://aace.org.uk/best-practice/las/> | Paramedic crewed with a mental health nurse, responding to patients requiring a mental health response. | Reduced ED conveyances from 55% to 24%, equating to around 30,000 fewer patients a year. |
| <https://aace.org.uk/initiatives/frailty-response-line-hull-east-riding/> | Frailty response line manned by a consultant geriatrician accessible by paramedics and community care staff for frail residents. | 175 patients were referred between 23^rd^ March and 31^st^ May 2020. 35 had suspected or confirmed COVID-19. Of the 35 suspected / confirmed COVID-19, 29 remained at home with a plan initiated. Of the 140 non-COVID-19, 82 remained at home through joint care planning. |
| <https://aace.org.uk/initiatives/leeds-paramedic-primary-rotation/> | Specialist paramedics rotated across 15 GP surgeries conducting home visits. They also rotated through the 999-control centre and provided frontline operational duties to safely manage patients away from the Emergency Department. | Post rotation, the paramedic intervention group demonstrated an increase in appropriate non-conveyances by 35%. Post placement, the cost per appropriate non-conveyance for intervention paramedics was a mean of £509.42 versus £1124.41 for the control group. This represents a mean saving of £615 per appropriate non-conveyance. |
| <https://aace.org.uk/initiatives/falls-rapid-response-service-frrs/> | Paramedic and occupational therapist worked collaboratively to respond to any falls related calls for patients >60 years who had fallen at home. | Prior to the service only 25% of falls related jobs were not conveyed to hospital. Since the service was introduced, this figure has increased to 56%. Of the 626 patients not conveyed to hospital, 75% required onward referrals to other community teams. |
| <https://www.england.nhs.uk/publication/wokingham-paramedic-home-visiting-model/> | Integrated paramedic home visiting model with patients being seen at home by a paramedic to reduce GP workload. | 96 hospital attendances were avoided, 171 calls to NHS 111 saved, 58 pathway referrals, and 75 GP appointments saved. |
